# Variation of gene ratios in mock communities constructed with purified 16S rRNA during processing

**DOI:** 10.1038/s41598-024-61614-1

**Published:** 2024-12-30

**Authors:** Georges Mikhael Nammoura Neto, René Peter Schneider

**Affiliations:** 1 Department of Microbiology, Institute of Biomedical Sciences, University of São Paulo, Av. Professor Lineu Prestes, 1374, São Paulo, 05508-900 Brazil; 2 Department of Chemical Engineering, Polytechnic School, University of São Paulo, Av. Prof. Luciano Gualberto, Travessa 3, n. 380., São Paulo, SP CEP 05508-900 Brazil

**Keywords:** Applied microbiology, Microbiology, Microbial communities, Microbial ecology

## Abstract

16S ribosomal nucleic acid (16S rRNA) analysis allows to specifically target the metabolically active members of microbial communities. The stability of the ratios between target genes in the workflow, which is essential for the bioprocess-relevance of the data derived from this analysis, was investigated using synthetic mock communities constructed by mixing purified 16S rRNA from *Bacillus subtilis* (Bs)*, Staphylococcus aureus* (Sa)*, Pseudomonas aeruginosa* (Pa)*, Klebsiella pneumoniae* (Kp) and *Burkholderia cepacia* (Bc) in different proportions. The RT reaction yielded one copy of cDNA per rRNA molecule for Pa, Bc and Sa but only 2/3 of the expected cDNA from 16S rRNAs of Bs and Kp. The combination of Taq DNA Platinum polymerase with subcycling PCR (scPCR) produced uniform yields of approximately 70% for second strand PCR synthesis from all target cDNAs. The proportion between templates in multicycle PCR was best preserved after 10 cycle scPCR followed by cloning. With MiSeq sequencing, correct proportions for about two thirds of templates were recovered after 10 cycle scPCR with Taq Platinum. 30 cycles standard PCR (stdPCR) or scPCR proved particularly harmful to proportion data and should be avoided.

## Introduction

The identification of physiologically active organisms is of critical importance for advancing bioprocess and biofouling control technologies. Cultivation-based techniques traditionally employed for this purpose have been superseded by nucleic-acid based culture-independent approaches due to the impossibility of accurately mimicking in the laboratory the myriad of diverse microniches typical for these environments. Community characterization of prokaryotes is mostly based on the sequencing of the highly conserved 16S rRNA gene, which is rarely transferred horizontally^1,2^. In this gene, the intercalation of the variable sequence domains important for organism identification with highly conserved segments allows targeting the latter with a limited set of “universal” primers for selective amplification of the variable sections^2^. 16S rRNA gene sequences can be obtained from rRNA or rDNA. DNA generally does not allow to discriminate metabolically active bacteria from latent or dead non-lysed organisms because these cells contain the same number of target gene copies^3^. Genome-based 16S rRNA analysis is further complicated by the up to 15 slightly different copies of this gene present in microbial genomes^2^. 16S rRNA is better suited for targeting the metabolically active prokaryotes because its amount is proportional to the number of ribosomes in a cell; actively metabolizing cells produce significantly more rRNA than slowly metabolizing or dormant ones^4^. It´s rapid degradation after cell lysis eliminates the likelihood of interference from extracellular molecular targets^4^. rRNA is usually transcribed from a single 16S rDNA gene even in cells harboring multiple copies of the gene, which greatly simplifies matching a sequence to an organism in genetic studies. Nogales et al.^5^, and Zakrzewski et al.^6^ reported a more accurate representation of community dynamics in bioreactors when the analysis was started from 16S rRNA than from 16S rDNA. Working with RNA is, however, more challenging as the molecule is less stable than DNA.

A typical rRNA workflow begins with the extraction and cleanup of the molecule from the sample. Enzymatic RT/PCR reactions convert the labile rRNA into the corresponding more robust cDNA prior to selective amplification of the genes of interest by PCR protocols. Individual templates in the final amplicon mixture are separated by cloning prior to sequencing or the whole sample is sequenced by one of the recently developed NGS protocols. Bioinformatics processing routines produce a final list of curated sequences from the raw sequencing data. Information of bioprocess relevance will be obtained by this workflow if (a) target molecules are quantitatively extracted from all cells and cleaned up with minimal losses and (b) when all relevant target genes are recovered at the end of the procedure in ratios close to those in the original extract. Incomplete lysis or losses during cleanup may lead to underrepresentation of process-relevant organisms in the extract^7^. Interferences in enzymatic post-extraction processing that might change gene ratios or composition include inhibition of enzymatic activity by extract components such as humics^8^, target-specific variability of primer binding and elongation kinetics^9^ and the introduction of spurious gene sequences into the gene pool by mutations or formation of heteroduplexes^10^. High-throughput sequencing is prone to platform-specific processing errors^11^.

The literature contains a wealth of information about the mechanisms that contribute to the preferential amplification of target genes in DNA mixed template stdPCR workflows. To our knowledge, no such information is available for 16S rRNA processing workflows. Here we present a systematic evaluation of how the individual 16S rRNA post extraction processing operations RT and PCR (standard and subcycling) affect the proportion of different 16S rRNA genes representing individual community members. Mock communities were established by mixing rRNA from pure cultures to eliminate variables associated with the extraction and purification of rRNA. Since published cloning data is widespread in the literature, analysis included rRNA processing workflows finalized either by cloning or NGS with the MiSeq protocol.

## Results

### cDNA synthesis (RT) from pure culture RNA extracts

**Figure 1:**
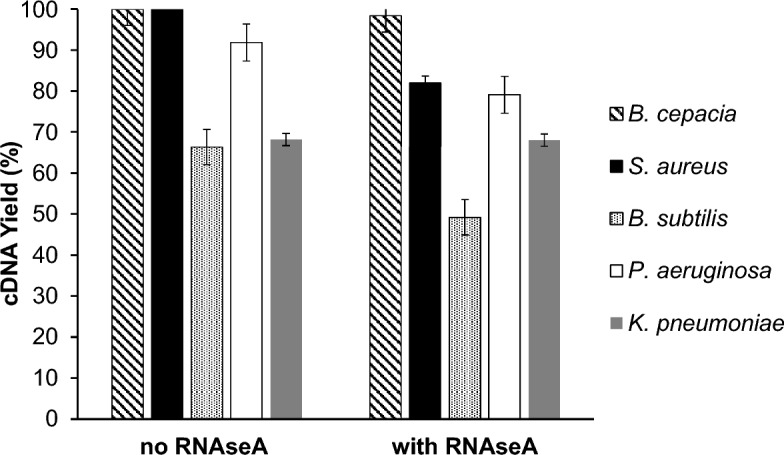
Single strand cDNA yield by RT. Means and range from duplicate analysis of samples. A yield of 100% would be equivalent to 1 cDNA copy/rRNA molecule.

Yields of cDNA in RT were evaluated only for pure culture 16S rRNA because of the impossibility of separating the individual cDNAs from a mixed template sample. RT showed excellent reproducibility and yields of > 92% and 66–68% for rRNAs of Bc, Sa, Pa and Bs as well as Kp, respectively. (Fig. 1).

**Figure 2:**
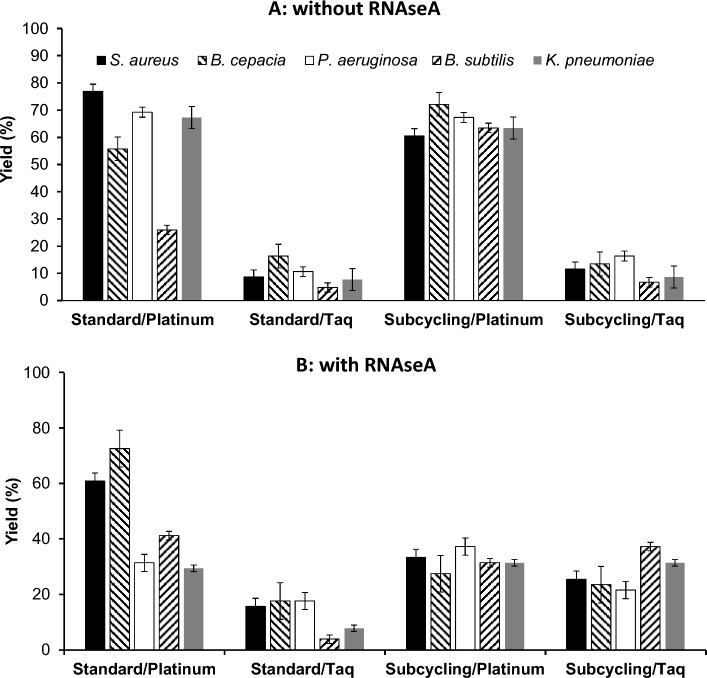
Second strand synthesis in RT-PCR of cDNA without (**A**) and with (**B**) RNAseA pretreatment. Standard: standard thermocycling (stdPCR), Subcycling thermocycling (scPCR). Platinum: Platinum DNA polymerase, Taq: taq DNA polymerase. A yield of 100% would correspond to one second strand synthesized for every single strand template present initially in the reaction.

Evaluations of yields at the next processing stage, the synthesis of the 2nd DNA strand in cDNA, included assays by stdPCR or scPCR with either Taq or Taq Platinum polymerases and with or without addition of RNAseA (Fig. 2). The reaction yield was measured in %, whereby 100% corresponded to a completed 2nd strand for each single strand template. The best performing combination of subcycling (scPCR) Taq platinum without RNAseA produced 2nd strand yields slightly above 70%, which were statistically similar for 8 of the 10 template pairs (Fig. 2, Table S1, suppl. info). RNAseA addition reduced average 2nd strand yield in assays with Taq Platinum but increased it slightly when Taq was employed.

### Effect of stdPCR cycles on the ratio between templates

#### Proportionality after cloning

Because of the high amount of labour involved, recovery of proportionality information by cloning was investigated only for a few mock communities. The proportions of templates in the starting sample were preserved relatively well after 10 cycles of stdPCR but not after 30 cycle stdPCR (Table 1). Proportionality change of Pa and Sa 16S rDNA after 30 cycle stdPCR depended on consortium composition. The ratio of Pa genes in consortium 1 was reduced to 1/7 but it was almost quadrupled in consortium 3. Trends were opposite for the ratios of genes of Sa which were halved in the consortium 3 and doubled in consortium 1. The proportions of Bs after 30 cycle stdPCR were not as affected. Cloning by itself preserved template ratios of the different cDNAs (Table 1).

**Table 1:** Proportion of templates of individual community members after 10 and 30 stdPCR cycles.

Initial ratio	stdPCR	Total clones	% *S. aureus*	% *P. aeruginosa*	% *B. subtilis*
			Proportions in final library (%)		
After full rRNA processing: RT/stdPCR → multicycle cDNA stdPCR → clone library					
1:1:1	10 cycles	105	38 (=)	28 (=)	30 (=)
1:1:1	30 cycles	106	66 (↑)	5 (↓)	30 (=)
8:1:1	10 cycles	104	75 (=)	15 (↑)	9 (=)
8:1:1	30 cycles	110	40 (↓)	37 (↑)	16 (↑)

Initial ratio	Total clones	% *S. aureus*	% *P. aeruginosa*	% *B. subtilis*	
		Proportions in final library (%)			
After cloning only: starting consortia obtained by mixing cDNA from the different organisms at the desired proportions					
(1:1:1)	132	32 (=)	36 (=)	33 (=)	
(2:1:1)	115	53(=)	23 (=)	24 (=)	
(1:8:1)	145	8(=)	85 (=)	9 (=)	

Consortia were assembled with purified rRNA from *S.* *aureus* (51% G + C), *P.* *aeruginosa* (54% G + C) and *B.* *subtilis* (55% G + C) in the proportions of 1:1:1 (consortium 1), 2:1:1 (consortium 2) and 1:8:1 (consortium 3). Arrows: changes relative to proportion in starting rRNA mix. = : no change, within ± 5% of that in starting mix; ↑ higher (above + 5%), ↓ lower (below − 5%).

#### Proportionality of templates after MiSeq sequencing

**Figure 3:**
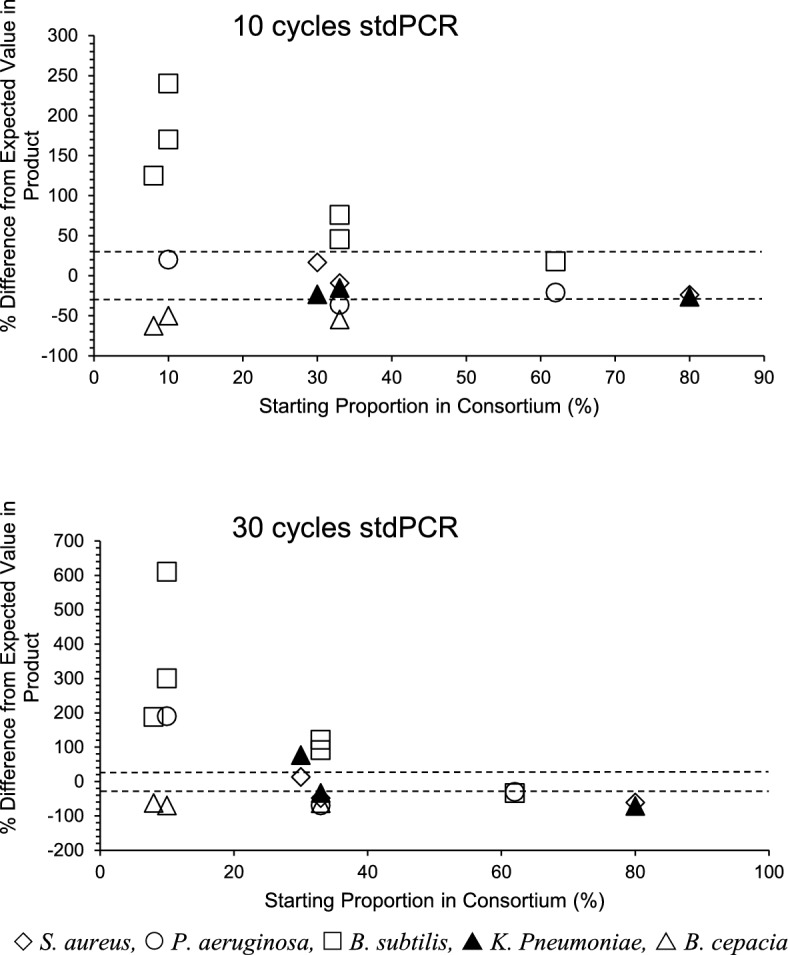
Percentage difference relative to the initial ratios one of template proportion in product of 10-cycle and 30-cycle stdPCR in the amplification of artificial 3-membered consortia. Dashed horizontal lines indicate the ± 30% interval where variations were not considered significant.

**Figure 4:**
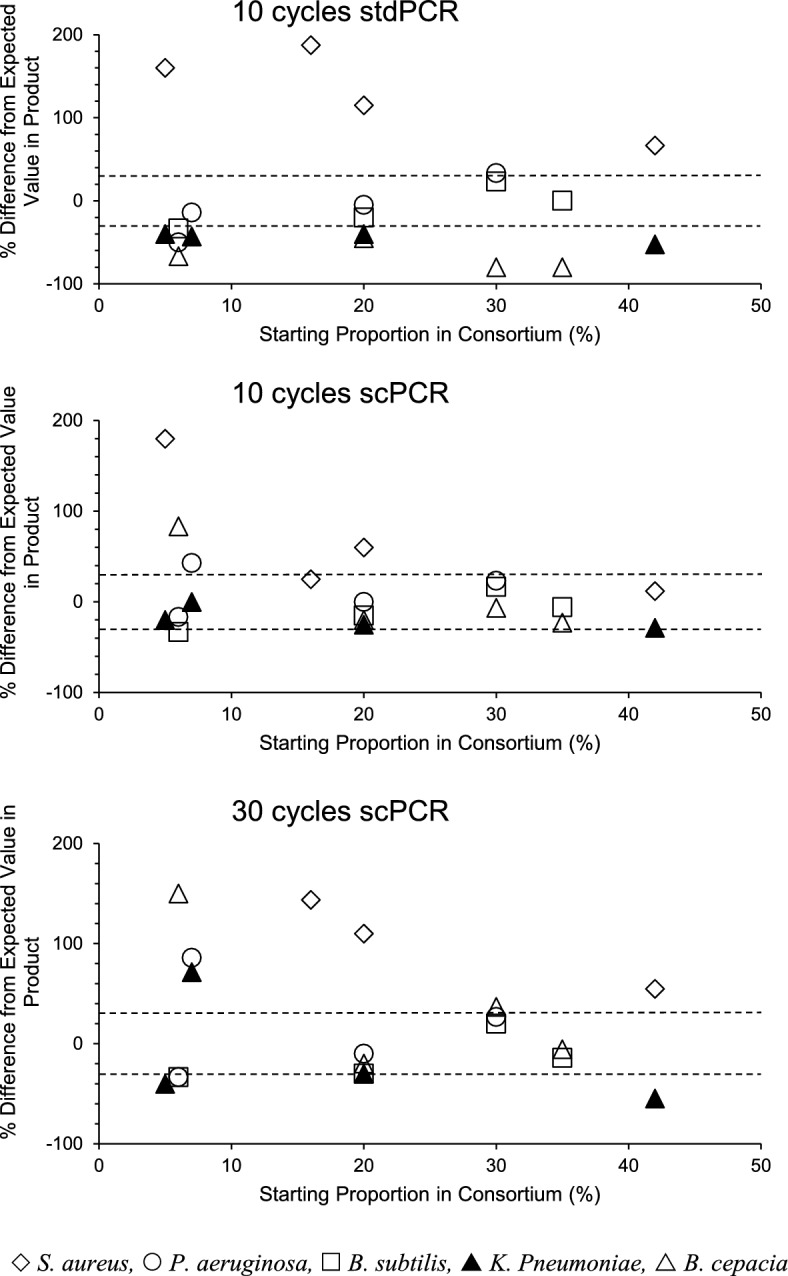
Percentage difference relative to the initial ratios one of template proportion in product of 10-cycle and 30-cycle stdPCR and scPCR in the amplification of artificial 5-membered consortia. Dashed horizontal lines indicate the ± 30% interval where variations were not considered significant.

MiSeq deep sequencing significantly modified the proportions of genes in 3-membered consortia after both 10- and 30-cycles stdPCR (Table S2 suppl. info, Figs. 3 and 4). Of the 36 genes assessed in these experiments, only 9 and 1 were recovered within ± 30% of their initial ratios after 10 and 30 cycle stdPCR, respectively (Table S2 suppl. info, Fig. 3). Template proportion was better preserved in 5-member consortia after scPCR where acceptable amplification performance was obtained for 75% (10 subcycles), 25% (10 stdPCR) and 40% (30 subcycles) of templates (Table S2 suppl info, Fig. 4).

The proportionality of individual genes in the final product depended on the gene mix in the consortium. Bs genes were generally overamplified in 3-member consortia, but mostly recovered at the expected ratios in 5-member consortia. The opposite occurred with Sa templates. Bc sequences were diluted out in the final product of 3-member consortia and of 5-member consortia submitted to 10 cycles stdPCR, but most of the amplicons from this organism were within the acceptable range when 5-member consortia were amplified by 10 cycles scPCR. Templates from Pa were mostly recovered within the expected range after 10 cycles, but not after 30 cycles stdPCR and scPCR in both 3- and 5- membered consortia. Amplification products of Kp were recovered within the expected range after 10 cycle stdPCR in 3-member consortia and after 10 cycle scPCR in 5-membered ones, but 30 cycle amplification produced a significant modification of the frequency of templates from these organisms.

## Discussion

This is the first study of an rRNA-based community characterization workflow where the impact of individual unit operations on the ratio of templates in a sample was investigated. RT yields of cDNA were close to 100% for rRNA from Pa, Bc and Sa but only about 67% for rRNA from Bs and Kp. scPCR with Taq DNA Platinum produced double-stranded cDNA with a yield of 60–70% for all single strand rRNAs. Restricting to 10 cycles in the scPCR protocol was essential for recovering a product with the ratios of most genes within ± 30% of those in the starting sample. Cloning per se did not affect the proportion of genes, but biases of MiSeq NGS changed the ratio of amplicons, particularly for those present in lesser proportion in the mix. Similar investigations with mock communities of purified 16S rDNA also failed to recover in the final amplicon product abundances of numerically important OTUs at genus or species level equal to those in the starting sample^7,12,13^. Some genes were overrepresented by more than one order of magnitude, whilst the sequences of others were lost, but genes representing > 5% of the templates in the starting mixture were always detected even in reactions with strong PCR biases. The changes in the composition of templates were generally of lesser importance at the taxonomically less stringent phylum level^14^, but even in these cases minimization of PCR cycle number was recommended to reduce the impact of chimeras and polymerase errors^15^. Interference by organic and inorganic substances including humic compounds, calcium ions, phenol, ethanol, urea, salt bile, sodium dodecyl sulphate or EDTA^16^ were not of relevance in this work, where assays were started with rRNA purified from pure cultures. In the following sections the performance of each individual workflow processing step will be discussed.

rRNA as a starting point for community analysis not only offers the advantage of targeting specifically the metabolically active cells, but it is also less affected by the multiplicity of 16S rRNA genes in genomes. P. aeruginosa, S.aureus, B. cepacia, K. Pneumoniae and B. Subtilis used in this work carry 4,5,6,8 and 10 copies of their respective 16S rRNA operons in their genomes with a relatively small intragenetic heterogeneity (< 1%^17^,). Multiple 16S rRNA genes would affect the proportionality data only if their sequences there sufficiently divergent for the respective rRNAs to be allocated to different gene pools, provided these divergent 16S rRNAs were transcribed simultaneously in the cell. Very little information is available about the regulation of transcription of genetically different 16S rRNA genes in organisms harboring such operons. The few studies on this topic suggest that differential transcription of 16S rRNA genes occurs when cells need to fine-tune their physiological response to environmental conditions. For example, different ribosomal RNA operons were expressed in E. coli in different growth conditions to optimize protein synthesis^18^. The 16S rRNA pool of such cells would, therefore, consist mainly, if not entirely, of material transcribed from genes with the same sequence, but the 16S rRNA gene sequence of the rRNA could change depending on the physiological status of the cell. Proportionality information would not be affected by such a change in 16S rRNA gene expression, provided the divergent gene sequence is recognized as originating from the same organism.

RT of rRNA from Bc, Sa and Pa produced 1 copy of cDNA for each 16S rRNA template. The recovery of only about 2/3 of the expected product in RT of 16S rRNA from Bs and Kp would have led to the suppression of these genes in the product gene pool if only 1/3 of each rRNA molecule was converted into cDNA. The recovery of the genes from these organisms in the final product, however, suggests that 2/3 of the rRNA templates were fully transcribed into cDNA. The reproducibility of the RT reactions in our study was rather good when compared to the tremendous increase in variability of RT/PCR when target concentration was reduced from 50 to 12.5 ng/μl reported by Bustin et al.^19^. One or several of the many undesired side reactions of RT^20^ may be responsible for the low cDNA yield in RT of some rRNA molecules: (1) RNA-dependent DNA polymerase activity (classical RT activity, desired); (2) DNA-dependent DNA polymerase activity (competes with PCR polymerase, but less effective and thus undesired); (3) terminal nucleotidyl transferase-like activity (TdT) and/or non-template directed nucleotide addition (both undesired); (4) RNaseH activity, if the RT was not mutated to remove it (the superscript III enzyme used in this work carried such a mutation^21^); (5) strand transfer and displacement ability (undesired) and (6) generic nonspecific capacity to attach to nucleic acid strands (undesired). Secondary rRNA structures that cause polymerases to slow down or stop the reaction altogether may be partly responsible for the large differences of RT yields^22^ particularly in mixed template 16S rRNA RT, where the same primers are used to amplify a wide range of targets of often unknown sequence variations. The RT reaction with mRNA or viral RNA can be influenced by background RNA^23^, by primer type (gene specific or random hexamer) and concentration. RT yields varied > 19-fold in the conversion of different RNAs with the same RT enzyme^24,25^. Yields with different RT enzymes varied by up to 100 times depending on the target RNA^26,27^. Unfortunately, the Superscript III enzyme employed here was not evaluated in these studies.

Taq DNA polymerase generally produced significantly smaller yields of second strand DNA than the improved Taq Platinum. The scPCR was essential for ensuring the production of similar amounts of 2nd strand DNA from the five 16S rRNA gene targets. As observed for the RT reaction, lower than expected scPCR yields of 60–70% did not result in information loss, suggesting that scPCR synthesis was completed for each target. The uniform amplification of different genes with varying G + C content was favored in the scPCR protocol by the alternation between low and high temperatures in the annealing/elongation stages^28^. Second strand synthesis was not improved by addition of RNAseA to degrade 16S rRNA after completion of the RT reaction as proposed by Kitabayashi and Esaka^27^. 16S cDNA yields after combined RT/PCR varied from 66 to 73%^29^ to 91% for *Salmonella typhimurium*^30^ and 94.4–115.4% for 8 different lactic acid bacteria^31^. RT efficiency varied up to 90-fold depending on the choice of reverse transcriptase, priming strategy, and assay volume in a comparison of 9 cDNA kits and 12 qPCR kits for RT/PCR of eukaryotic mRNA^32^. Bustin & Nolan^33^ considered mRNA RT/qPCR a paradigm for the lack of reproducibility of molecular biological methods, primarily because of the variability of the RT reaction.

Because of its repetitive nature, PCR protocols are decisive for quality and quantity of the final products of the workflow. Mixed template PCR are carried out in an exceedingly complex and variable reaction mix. Nucleic acids transition between double and single strand configurations. The concentrations of nucleotides decrease and that of nucleic acids increase with each cycle. Amounts of partially amplified fragments that compete with primers for binding sites increase with each PCR cycle. For PCR steps to be successful, it is essential that the kinetics of desired primer binding and extension be favoured over those of the interfering processes. Denaturation at the start of PCR is least prone to interferences which occur primarily during primer binding in the annealing phase and extension.

Annealing is the workflow step where the primers attach to their targets. Assay temperature in this period is maintained at a value significantly below that used for denaturation or elongation for the time required for the saturation of all target sites with minimal undesired priming events. Primer access to the binding site may be obstructed by attached interfering molecules, by secondary ssDNA conformations or when the target region of ssDNA is blocked due to interactions with other DNA or RNA^34^. Primer binding to and elongation from non-target sites produces waste products that may interfere in the following cycles of amplification by formation of undesired DNA:DNA heteroduplexes^35^. Addition of DMSO may help destabilize undesired interactions^35^. Heteroduplex formation can be prevented with a reconditioning PCR, where the mixed-template product is diluted tenfold in the original PCR mix and then subjected to a 3-cycle re-amplification^36^.

Primers become incorporated into the product strand, their concentration in solution decreases and that of potential binding sites increases with each PCR cycle. The success of the primer interaction with its targets particularly in later stages of multicycle PCR depends on optimal fit. Although the primers for 16S rRNA-based community analysis target the evolutionary conserved sections of the gene, they still need to be designed to recognize a wide range of phylogenetic targets with slightly differing nucleotide sequences. Degeneracies in specific nucleotide positions such as those between A and C or T and C in the primers 27F and 1492R, respectively^37^ did not prevent mismatches. Single mismatches in the last 3–4 nucleotides from the 3′ end of a primer aborted amplification whilst negative effects due to mismatches from the 5th base onward from the 3′ end of the primer could be partially or fully compensated by lowering the annealing temperature by up to 7 °C^38^. Sipos et al.^39^ reported no bias in the amplification with perfectly matched 27F 16S rDNA primers of 16S rDNA from model communities produced by pairwise combination of *Aeromonas hydrophila*, *Bacillus cepacia*, *Bacillus subtilis* and *Pseudomonas fluorescens.* With the 63F primer, that harboured 3 mismatches towards the 5´end for *B. cepacia* and *B subtilis*, preferential amplification of the perfectly matching sequences of *A. hydrophila* and *P. fluorescens* occurred^39^. More importantly, no product was detected from mismatched DNA below a concentration threshold. Differences in stdPCR and scPCR yield between mismatched and fully matching primers were not important at low annealing temperatures but became substantial at higher annealing temperatures.

GC rich primers have a higher melting temperature than AT ones. The ternary polymerase-primer-template complex and nascent strand extension increase the stability of the primer/target bond at the annealing temperature. The minimal number of additional nucleotides that need to be added to a primer to increase the melting temperature above that used for elongation varies depending on primer sequence, annealing and elongation temperatures and nucleotide sequence of the portion that will be complemented (Table 2). In our case, the number of additional nucleotides required after primer binding for the stabilization of the primer/target complex by new hydrogen bonds provided by additional incorporated nucleotides varied from a low of 17 for primer 909r in Kp to a high of 35 in Bc with primers 27F and 1401r (Table 2). The time required for this elongation would be of the order of a few seconds, considering a 1000 bp/min nucleotide incorporation rate typical for polymerases at the optimal elongation temperature.

**Table 2:** Number of incorporated nucleotides required after annealing of primers to a length where the melting temperature exceeds the elongation temperature of 72 °C.

Required number of nucleotide for stabilization (downstream primer)				
*S. aureus*	*B. cepacia*	*P. aeruginosa*	*B. subtilis*	*K. pneumoniae*
Primer 27f., M13f.: annealing temperature 62.5 °C				
30	35	26	19	21
Primer 1401r, M13r: annealing temperature 62.5 °C				
30	35	27	28	31
Primer 338F1, F2 and F3: annealing temperature 55 °C				
27	21	29	31	24
Primer 909r: annealing temperature 55 °C				
33	31	33	32	17

The extension time of 100 s used in this work would have sufficed for complete duplication of the templates at a nucleotide incorporation rate of 1000 bp/min. Modification of proportions between different genes in the final amplicon mixture was caused by differences in the kinetics of amplification as reported for two membered consortia by Polz & Cavanaugh^40^ and for more diverse DNA template mixtures^15,28^. Taq platinum amplification efficiencies of 78.5% for sequences with 45% G + C dropped to 43.3% for sequences with 78% G + C, templates high in G + C required addition of DMSO for efficient amplification^35^. G + C of the 16S rDNA genes in this study varied from 51% to 56.5%. Bs 16S rDNA (55% G + C) was preferentially amplified in 11 of 12 3-membered consortia, but only in 3 of 12 5-membered consortia. DNA from another G + C rich organism, Kp (56% G + C), was recovered at or close to the expected proportions in most 3-membered consortia, but often overenriched in 5-membered ones. DNA from Sa with the lowest G + C (51%) was often enriched in 5 membered consortia and diluted in about half of the 3-membered communities and enriched in the other half. These results suggest that G + C content alone is not the main driver for preferential amplification.

Homoduplex formation by association of complementary single stranded DNA of dominant templates, which removes amplification targets from the reaction^9^, may explain the better amplification of the dominant G + C poor Sa genes in consortium 3 but not in the other 4 3-membered consortia and in none of the 5 member consortia. Techniques to minimize PCR biases for heterogeneous template mixtures include the emulsification of the PCR reaction with silicone oil^41^ and the reduction of the number of cycles in the PCR reaction^9^. Inhibition of the initial PCR steps due to the interference by segments outside the amplified sequence was identified as a cause for PCR bias by Hansen et al.^42^. Wu et al.^53^ reported significant differences in community structure after amplification of the same DNA extract with different polymerases.

Inhibition of PCR reaction by active RT enzymes protected from high temperature denaturation by association with rRNA is of relevance for the recovery of rare targets^18^. The significantly better maintenance of proportions of individual templates in the end product of 5-member consortia after 10 cycle scPCR was remarkable. Subcycling essentially interrupts the annealing phase with intermittent elongation phases. Instead of annealing at 62.5 °C for 1 min and extension at 72 °C for 100 s, subcycling switched 4 times between annealing at 60 °C for 1 min and extension at 65 °C for 1.5 min, which helped mitigate the interference of secondary structures on elongation^12^.

MiSeq sequencing by itself may contribute to skewing template proportions as revealed by the comparison of 16S rRNA target proportionality recovery for three membered consortia after 10 cycle standard RT/PCR by cloning (Table 1) and MiSeq (Fig. 3). This effect was more pronounced for 16S rRNA targets present in low proportion (Fig. 3).Potential bias mechanisms in the standardized manufacturer-specific MiSeq workflow are entirely different to those of 16S rRNA RT/PCR. Whilst 16S rRNA RT/PCR biases are sensitive to the sequences of the 16S rRNA primer binding sites, those of MiSeq are not. Potential MiSeq biases are related to (1) the proprietary chemistry employed for tagging and attaching the target DNAs to the supports from where they will be sequenced, (2) to the PCR procedures used to augment the population of the attached target genes and (3) sequencing biases. MiSeq PCR primers are directed at the linkers used to attach the target genes to the supports. Both linker attachment chemistry and linkers are identical for all different 16S rRNA target genes selected for sequencing. MiSeq biases for the mechanisms (1) and (2) can be significantly reduced to below 0.4% by assay optimization^43^. The major error source in type (3) biases are substitution type miscalls that lead to incorrect base assignment in the first 10 bp and in the last 50 bp of the reads^44^. These can be reduced by 93% with the substitution error correction strategies adopted in this work^44^. Variable regions of the 16S rRNA gene that harbor gene motifs prone to sequencing bias^45^ will be analyzed less efficiently and thus become diluted in the product mix. Next generation sequencing requires extensive raw data processing before producing a final output. The reduction of the raw data error rate by bioinformatics strategies for identifying bad sequence reads in combination with the correction of bad base calls reduced but did not eliminate wrong 16S rRNA sequence allocations^46^.

Cloning provided the best recovery of proportionality information with the mock 16S rRNA communities particularly when it was conducted directly from cDNA without prior PCR amplification. The long 16S rRNA gene segments used here for cloning were advantageous for species identification, but Huber et al.^47^ reported better representation of biodiversity in cloning libraries with short 16S rDNA gene segments of 100 bp and 400 bp segments than in those with the longer 1000 bp gene sections. The numerically dominant clone sequences were recovered in all three clone libraries^47^. The lower efficiency of the long gene sections was attributed to a combination of polymerase dissociation, cloning bias and mispriming that reduced the efficiency of amplification of these templates.

The measurement of the relative size of the populations of active microbial species is of utmost relevance to mixed culture bioprocess design and optimization. Such proportion data are essential for establishing a link between process efficiency and community structure and response, but these data are rarely measured. In this work we demonstrated that a protocol based on 16S rRNA extraction followed by RT/scPCR with amplification cycles restricted to 10 allows the recovery of meaningful information about the proportion of the active fraction of metabolically important organisms in mixed microbial communities. The best representation of proportions of templates was obtained after cloning, a very laborious and time-consuming technique, which has been superseded by modern NGS techniques because of their higher throughput and lower cost. The data produced with NGS at the optimized conditions were of marginally inferior quality to those obtained by cloning, but still acceptable for process analysis and optimization. The proportionality analysis method presented here will contribute to the improvement of strategies for the optimization and control of mixed culture biotechnology.

## Methods

### Mock communities

Stock cultures of Bs (ATCC 6633; NCBI 703612), Sa (ATCC 6538; NCTC 10,788), Pa (ATCC 15,442; NCBI 1424337), Kp (MGH 78,578; NCBI 272620) and Bc (ATCC 25,416; NCBI 983594) cultured in TSB and stored at 4 °C were grown in TSB on a rotary shaker at 37 °C and harvested at mid log phase by centrifugation (16000 g, 5 min, 4 °C). The pellets were stored at − 80 °C until extraction by the method of Nammoura Neto et al.^48^. Co-extracted DNA was removed with Turbo DNA-free™ (Thermo Scientific), rRNA was purified with the RNeasy MinElute Cleanup kit (Qiagen, Hilden, Germany) and quantified with the Nanodrop® ND-1000™ spectrophotometer (Thermo Scientific Waltham, Massachusetts, USA). Mock communities were produced by mixing purified 16S rRNA from the pure cultures in different ratios as shown in Table 3.

**Table 3:** Composition of mock communities. G + C was determined with GC-Profile^49^.

	*Staphylococcus aureus*	*Burkholderia cepacia*	*Pseudomonas aeruginosa*	*Bacillus subtilis*	*Klebsiella pneumoniae*
G + C:	51%	52%	54%	55%	56%

Consortia*	ng pure culture rRNA				
1	5000	–	5000	5000	–
2	20,000	–	5000	40,000	–
3	40,000	–	5000	5000	–
4	–	5000	–	5000	5000
5	–	40,000	–	5000	20,000
6	–	5000	–	5000	40,000
7	5000	5000	5000	5000	5000
8	40,000	5000	5000	5000	40,000
9	5000	40,000	40,000	40,000	5000
10	2000	40,000	5000	40,000	5000

### RT

Components and conditions used in RT reactions are listed in Tables 4 and 5. The cDNA product was treated with RNAseA immediately after RT. rRNA, sscDNA and dscDNA were quantified with the DeNovix DS-11 (DeNovix, Wilmington, Delaware, EUA) spectrophotometer. Readings were corrected using blanks without added sample.

**Table 4:** Composition of amplification assays.

Additive	RT (cDNA)	RT-PCR 2nd strand	stdPCR scPCR	MiSeq PCR	p-Gem® Easy-T Vector
Primers	1401R 20 mM, 1 μl	27F/1401R, each 10 mM, 0.25 μl	cloning: 27F/1401R, each 10 mM, 0.25 μl MiSeq: 338F1/909R 338F2/909R 338F3/909R each 10 mM, 0.25 μl	Proprietary PCR Primer Cocktail^c^ 5 μl	M13F/M13R each 10 mM, 0.25 μl
Polymerase	1 ml SuperScriptIII^a^	2U Taq Platinum^a^ or 2U Taq^b^	2U Taq Platinuma or 2U Taqb	Proprietary PCR Master Mixc 25 μl	1U Taq^b^
MgCl2	NA	50 mM (2 µl)	50 mM (2 µl)	ND3)	50 mM (0.85 µl)
dNTP Mix^a^	10 mM (1 µl)	10 mM (1.25 µl)	10 mM (1.25 µl)	ND3)	10 mM (0.63 µl)
Buffer	4 µl	10X Pfu Buffer (2.5 μl)	10X Pfu Buffer (2.5 μl)	ND3)	10X Pfu Buffer (2.5 μl)
DTT	0.1 M (1 µl)	–	–	–	–
Starting material	2μgrRNA	2μlRT product	2μlRT product	20 µL	Colony sample collected with sterile platinum wire
Ultrapure water^a^ up to final volume of		25 μλ	25 μλ	–	25 μλ
Reference	Manufacturer	stdPCR^50^ scPCR^28^	stdPCR^50^ scPCR^28^	Manufacturer	Manufacturer

^a^Invitrogen Life Technologies, São Paulo, Brazil.

^b^Sinapse Inc., São Paulo, SP, Brazil.

^c^Illumina, San Diego, California, USA.

Primers:

27 F: 5′-AGA GTT TGA TCM TGG CTC AG-3′^37^.

1401R: 5′-CGG TGT GTA CAA GGC CCG GGA ACG-3′^51^.

338F1: 5′-CCT ACG GGR GGC AGC AG-3′^7^.

338F2: 5′-ACW YCT ACG GRW GGC TGC-3′^7^.

338F3: 5′-CAC CTA CGG GTG GCA GC-3′^7^.

909R: 5′-CCG TCA ATT YTT TTR AGT-3′^7^.

M13F: 5′-CGCCAGGGTTTTCCCAGTCACGAC-3′ (Promega cloning vector).

M13R: 5′-TTTCACACAGGAAACAGCTATGAC-3′ (Promega cloning vector).

**Table 5:** Temperature programs implemented in the Mastercycler Gradient thermal cycler (Eppendorf AG, Hamburg, Germany).

Reaction	RT (cDNA)	RT-PCR 2nd strand	stdPCR	scPCR	MiSeq PCR	p-Gem® Easy-T Vector
Initial denaturation	–	–	94 °C 5 min	94 °C 5 min	98 °C 10 s	94 °C 5 min
Cycle denaturation	–	either stdPCR or scPCR	94 °C for 30 s	94 °C for 20 s	98 °C 10 s	94 °C for 30 s
Annealing	65 °C, 5 min; ice for 1 min	either stdPCR or scPCR	27F/1401R: 62.5 °C 60 s 338F1/909R 338F2/909R 338F3/909R: 55 °C 20 s	$$\begin{array}{*{20}c} {4 \times 60\;^\circ {\text{C}}/60\;{\text{s}}} \\ \updownarrow \\ {65\;^\circ {\text{C}}/90\;{\text{s}}} \\ \end{array}$$	60 °C/30 s	60 °C/30 s
Elongation	50 °C/45 min	either stdPCR or scPCR	27F/1401R: 72 °C/100 s 338F1/909R 338F2/909R 338F3/909R: 72 °C/30 s	$$\begin{array}{*{20}c} {4 \times 60\;^\circ {\text{C}}/60\;{\text{s}}} \\ \updownarrow \\ {65\;^\circ {\text{C}}/90\;{\text{s}}} \\ \end{array}$$	72 °C/30 s	72 °C/60 s
Inactivation or final elongation	70 °C/15 min	either stdPCR or scPCR		65 °C/5 min	72 °C/5 min	72 °C/5 min
Number of PCR cycles	–	2	10 or 30	10 or 30	15 (manufacturer)	40

Temperature ramps were 1 °C/s.

### RT-PCR

Second strand synthesis RT-PCR assay components and conditions are summarized in Tables 4 and 5.

### PCR

Primers, assay components and temperature cycling routines employed for stdPCR and scPCR are summarized in Tables 4 and 5. 16S rRNA gene sequences of the study organisms and the target sites for primer binding were provided in the supplementary information. Whilst the entire 16S RNA gene was amplified for cloning experiments, MiSeq analysis required shorter product segments to allow for direct processing by the sequencer. MiSeq amplicons covering the V3–V5 hypervariable region^7,52^ of the 16S rRNA gene were produced using three modified forward and one reverse primer aiming for greater coverage of bacterial diversity (Tables 4 and 5). DNA for MiSeq sequencing was produced in three parallel 10 cycle stdPCR and scPCR reactions and the products were combined in a single tube for further processing. cDNA was purified with the GFX™ PCR kit DNA or Gel Band Purification Kit (Amersham Biosciences, UK) following manufacturer´s instructions, analyzed on 1% agarose gels and quantified with a Nanodrop® ND-1000 spectrophotometer. To obtain sufficient material for cloning, stdPCR and scPCR with 10 cycles were run in triplicate. The products were mixed, and the volume was reduced to 10μL in a vacuum concentrator prior to further processing. PCR or RT yields were determined from concentration measurements of the products of interest.

### Cloning and ARDRA

Target genes were cloned into the p-Gem® Easy-T Vector that was inserted into competent *Escherichia coli*-JM 109 cells for multiplication following manufacturer´s instructions (all Promega, Madison, WI, USA, Tables 4 and 5). ARDRA restriction digests were produced with five restriction enzymes: Hae III (GG/CC–CC/GG, Promega, Madison, WI, USA), Hha I (GCG/C–C/GCG, Promega, Madison, WI, USA), Rsa I (GT/AC–CA/TG, Biolabs, New England), Msp I (C/CGG–GGC/C, Biolabs-New England) and Bhs1236I (CG/CG, Fermentas, Mariland, USA). A 50 base pair DNA Ladder was used as molecular marker in agarose gels (Fermentas, Mariland, USA).

### Next generation sequencing

The PCR product mix was processed with the TrueSeq RNA Sample Prep v.2 kit for sequencing with MiSeq using the MiSeq Reagent v3 kit (Illumina, San Diego, California, USA, Tables 4 and 5). Raw data were preprocessed with the 16S Metagenomics App (basespace.ilumina.com), which uses Naïve Bayes as a taxonomic classification algorithm and the GreenGenes 13_5 version database. In addition, sequence analysis was performed with the QIIME software, together with the SILVA 138.1 version (97% similarity) and GreenGenes 13_5 version databases (97% and 99% similarity). The overlapping region for the V3–V5 hypervariable region was 29 bp, 21 bp smaller than recommended by the manufacturer. However, bioinformatics data processing was applied to filter out low-quality sequences (section informatics and database in supplementary information). Sequences with similarity of less than 50% in overlapping sections, fragments shorter than 585 bp and sequences comprising less than 0,005% of the total number of valid reads were excluded. The overall yield of valid reads was around 85% ± 1% for the different mock communities, which shows the reproducibility of the procedure.

### Quality criteria for proportionality recovery

Ratios of templates in the final product within ± 30% of their proportion in the starting sample were considered acceptable. This criterion accommodates the many potential sources of error whilst still delivering data of process relevance. For example, in the case of a sequence representing 50% of all templates in the sample, the 30% criterion would consider any value from 35 to 65% as acceptable. In the case of rarer templates, for example, for one that made up 2% of the population, values between 1.4 and 2.6% would be considered acceptable.

### Statistical analysis

Statistical comparison of means, where appropriate, was first performed with the ANOVA Single Factor variance tool (*p* = 0.05%). Pairwise comparison of means was performed with a Tukey-Cramer post hoc test at *p* = 0.05%.

## Supplementary Information

Supplementary Information.

## Data Availability

The datasets generated during and/or analysed during the current study are available from the corresponding author on reasonable request.
