# Supplementary material for: Variation of gene ratios in mock communities constructed with purified 16S rRNA during processing
Source: Sci Rep. 2024 Dec 30;14:31577. doi: 10.1038/s41598-024-61614-1 (PMC11686170; doi:10.1038/s41598-024-61614-1)
Supplement: Supplementary file 1 — Supplementary Information. [file 41598_2024_61614_MOESM1_ESM.docx]

**Supplementary Information:**

**Modification of Gene Ratios In Mock Communities Constructed with Purified 16S rRNA During Processing**

**Journal: Scientific Reports**

**Georges Mikhael Nammoura Neto**^1^, **René Peter Schneider**^2, *^

^1^Department of Microbiology, Institute of Biomedical Sciences, University of São Paulo, Av. Professor Lineu Prestes, 1374, 05508-900 São Paulo, Brazil

^2^Department of Chemical Engineering, Polytechnical School, University of São Paulo, Av. Prof. Luciano Gualberto, Travessa 3, n. 380. São Paulo – SP, Brazil. CEP 05508-900.

*corresponding author, e-mail: schneiderpqi@usp.br

**Table S1** Statistical analysis of second strand synthesis in RT-PCR of cDNA with and without RNAseA pretreatment. Reactions performed with Taq or Taq Platinum polymerases by the stdPCR or scPCR amplification protocols. Raw data are shown in Figure 3. Statistical comparison of mean values of all strains: ANOVA (p=0.05%). Pairwise comparisons: Tukey-Cramer post hoc test at p=0.05%: ≠: statistically different; = statistically similar. Sa: *Staphylococcus aureus*, Bc: *Burkholderia cepacia*, Pa: *Pseudomonas aeruginosa*, Bs*:*  *Bacillus subtilis* and Kp: *Klebsiella pneumonia.*

| **Comparison** | **With RNAseA** | | | | **Without RNAseA** | | | |
| --- | --- | --- | --- | --- | --- | --- | --- | --- |
|  | **PCR conditions** | | | | **PCR conditions** | | | |
|  | **std-PCR** | | **sc-PCR** | | **std-PCR** | | **sc-PCR** | |
|  | **Platinum** | **Taq** | **Platinum** | **Taq** | **Platinum** | **Taq** | **Platinum** | **Taq** |
| ANOVA | ≠ | ≠ | ≠ | ≠ | ≠ | ≠ | = | ≠ |
| mean Bc – mean Sa | ≠ | = | ≠ | = | ≠ | ≠ | = | = |
| mean Bc – mean Bs | ≠ | ≠ | = | ≠ | ≠ | ≠ | = | ≠ |
| mean Bc – mean Pa | ≠ | = | = | ≠ | ≠ | ≠ | = | ≠ |
| mean Bc – mean Kp | ≠ | ≠ | = | ≠ | ≠ | ≠ | = | ≠ |
| mean Sa – mean Bs | ≠ | ≠ | ≠ | ≠ | ≠ | ≠ | = | ≠ |
| mean Sa – mean Pa | ≠ | = | ≠ | = | ≠ | = | ≠ | ≠ |
| mean Sa – mean Kp | ≠ | ≠ | = | ≠ | ≠ | = | = | ≠ |
| mean Pa – mean Kp | ≠ | ≠ | ≠ | ≠ | ≠ | ≠ | ≠ | ≠ |
| mean Bs – mean Kp | = | ≠ | = | = | ≠ | ≠ | = | ≠ |
| mean Bs – mean Pa | = | ≠ | ≠ | = | = | = | = | ≠ |
| = means | 2 | 3 | 5 | 4 | 1 | 3 | 8 | 1 |

**Table S2** Effect of PCR conditions (cycle number and cycling program) on the proportion of templates of individual community members in consortia assembled with purified rRNA from pure cultures of B. *subtilis* (Bs), S. *aureus* (Sa), *P*. *aeruginosa* (Pa), *K. pseudomoniae* (Kp) and *B. cepacia* (Bc). Final PCR product analyzed by deep sequencing on a MiSeq platform. Data in the table are percentage difference between obtained and expected value after final analysis of sequencing data. Starting template proportion in consortia given in Table 1. PCR protocol: Standard: stdPCR; Subcycling: scPCR. Numbers in bold are those where the difference between expected and measured proportion remained within ±30%.

1. 3-membered consortia after full rRNA processing: RT/PCR → multicycle cDNA PCR → deep sequencing.

| **Consortium** | **PCR** | **Sa** | **Bc** | **Pa** | **Bs** | **Kp** |
| --- | --- | --- | --- | --- | --- | --- |
| 1 | Std 10 cycles | **-9,1** | - | -36,4 | 45,5 | - |
| 1 | Std 30 cycles | -48,5 | - | -69,7 | 121,2 | - |
| 3 | Std 10 cycles | **-23,8** | - | **20** | 170,0 | - |
| 3 | Std 30 cycles | -61,3 | - | 190 | 300 | - |
| 2 | Std 10 cycles | **16,7** | - | **-21,0** | 125,0 | - |
| 2 | Std 30 cycles | **13,3** | - | -30,6 | 187,5 | - |
| 4 | Std 10 cycles | - | -54,5 | - | 75,8 | **-15,2** |
| 4 | Std 30 cycles | - | -63,6 | - | 90,9 | -33,3 |
| 6 | Std 10 cycles | - | -50,0 | - | 240,0 | **-26,3** |
| 6 | Std 30 cycles | - | -70,0 | - | 610,0 | -71,3 |
| 5 | Std 10 cycles | - | -62,5 | - | **17,7** | **-23,3** |
| 5 | Std 30 cycles | - | -62,5 | - | -33,9 | -76,7 |

1. 5-membered consortia after full rRNA processing: RT/PCR → multicycle cDNA PCR → deep sequencing.

| **Consortium** | **PCR** | **% Sa** | **% Bc** | **% Pa** | **% Bs** | **% Kp** |
| --- | --- | --- | --- | --- | --- | --- |
| 7 | Sc 10 cycles | 60.0 | **-20.0** | **0** | **-15.0** | **-25.0** |
| 7 | Sc 30 cycles | 110,0 | **-20,0** | **-10,0** | **-30,0** | **-30.0** |
| 7 | Std 10 cycles | 115.0 | -45.0 | **-5.0** | **-20.0** | -40.0 |
| 8 | Sc 10 cycles | **11.9** | 83.3 | **-16.7** | -33.0 | **-28.0** |
| 8 | Sc 30 cycles | 54,8 | 150.0 | -33,3 | -33.0 | -55.0 |
| 8 | Std 10 cycles | 66.7 | -67.0 | -50.0 | -33.0 | -52.5 |
| 9 | Sc 10 cycles | 180.0 | **-6.7** | **23** | **17.0** | **-20.0** |
| 9 | Sc 30 cycles | 240,0 | 36,7 | **26,7** | **20.0** | -40.0 |
| 9 | Std 10 cycles | 160.0 | -80.0 | 33.3 | **23.0** | -40.0 |
| 10 | Sc 10 cycles | **25.0** | **-22.9** | 43.0 | **-5.5** | **0.0** |
| 10 | Sc 30 cycles | 143,8 | **-5,7** | 85,7 | **-14.0** | 71.4 |
| 10 | Std 10 cycles | 187.5 | -80.0 | **-14.0** | **0** | -42.9 |

Bioinformatics and database processing

A pre-treatment manual was implemented using the QIIME tool. The forward.fasta and reverse.fasta files generated by sequencing on the MiSeq were merged into a single file. Different similarity values were tested for the overlapping region, with the value of 50% allowing the pairing between the two sequences without significant sample loss.

- **join_paired_ends.py -p 50 -f <forward_reads.fastq> -r <reverse_reads.fastq> -o <output>**

NextGen software was used to filter sequences with a minimum size of 585bp. This step was applied with the aim of removing sequences that did not reach the size of the V3-V5 region. The generated file was then submitted to the commands below:

- **convert_fastaqual_fastq.py -c fastq_to_fastaqual -f <input.fastq> -o <output>**
- **split_libraries.py -b 0 -p -m <Fasting_Map.txt> -f <Fasting_Example.fna> -q <Fasting_Example.qual> -o <output>**

After this step, the OTUs were constructed using different databases as a reference for similarity and taxonomy. The databases used were SILVA version 123.1 (03/2016) and Greengenes version gg_13_5 (05/2013).

**• pick_closed_reference_otus.py –i <seqs.fna> -r <reference_16S.fasta> -o <output> -t <taxonomy_16S.txt>**

A second filtering step was adopted to exclude OTUs with very low abundance. Navas-Molinas (2013) and Bokulich (2013) recommend discarding OTUs with a number of sequences less than 0.005% of the total number of sequences.

- **filter_otus_from_otu_table.py –i <otu_table.biom> -o <filtered.biom> --min_count_fraction 0.00005**

The values obtained in the generated BIOM file were observed using the command below:

- **biom summarize-table -i <filtered.biom>**

To generate taxonomic diversity results and rarefaction curves for each sample analyzed, the command below was executed:

- **core_diversity_analyses.py -i <filtered.biom> -m <Fasting_Map.txt> -t <reference.tre> -e <total counts> -o <output> --suppress_beta_diversity**

**Black**: 16S rRNA full length

**Bold and underline:** 27F and 1041R binding sites

**STAPHYLOCOCCUS AUREUS ATCC 13883- 51% GC**

TTTATGG**AGAGTTTGATCCTGGCTCAG**GATGAACGCTGGCGGCGTGCCTAATACATGCAAGTCGAGCGAA

CGGACGAGAAGCTTGCTTCTCTGATGTTAGCGGCGGACGGGTGAGTAACACGTGGATAACCTACCTATAA

GACTGGGATAACTTCGGGAAACCGGAGCTAATACCGGATAATATTTTGAACCGCATGGTTCAAAAGTGAA

AGACGGTCTTGCTGTCACTTATAGATGGATCCGCGCTGCATTAGCTAGTTGGTAAGGTAACGGCTTACCA

AGGCAACGATACGTAGCCGACCTGAGAGGGTGATCGGCCACACTGGAACTGAGACACGGTCCAGACTCCT

ACGGGAGGCAGCAGTAGGGAATCTTCCGCAATGGGCGAAAGCCTGACGGAGCAACGCCGCGTGAGTGATG

AAGGTCTTCGGATCGTAAAACTCTGTTATTAGGGAAGAACATATGTGTAAGTAACTGTGCACATCTTGAC

GGTACCTAATCAGAAAGCCACGGCTAACTACGTGCCAGCAGCCGCGGTAATACGTAGGTGGCAAGCGTTA

TCCGGAATTATTGGGCGTAAAGCGCGCGTAGGCGGTTTTTTAAGTCTGATGTGAAAGCCCACGGCTCAAC

CGTGGAGGGTCATTGGAAACTGGAAAACTTGAGTGCAGAAGAGGAAAGTGGAATTCCATGTGTAGCGGTG

AAATGCGCAGAGATATGGAGGAACACCAGTGGCGAAGGCGACTTTCTGGTCTGTAACTGACGCTGATGTG

CGAAAGCGTGGGGATCAAACAGGATTAGATACCCTGGTAGTCCACGCCGTAAACGATGAGTGCTAAGTGT

TAGGGGGTTTCCGCCCCTTAGTGCTGCAGCTAACGCATTAAGCACTCCGCCTGGGGAGTACGACCGCAAG

GTTGAAACTCAAAGGAATTGACGGGGACCCGCACAAGCGGTGGAGCATGTGGTTTAATTCGAAGCAACGC

GAAGAACCTTACCAAATCTTGACATCCTTTGACAACTCTAGAGATAGAGCCTTCCCCTTCGGGGGACAAA

GTGACAGGTGGTGCATGGTTGTCGTCAGCTCGTGTCGTGAGATGTTGGGTTAAGTCCCGCAACGAGCGCA

ACCCTTAAGCTTAGTTGCCATCATTAAGTTGGGCACTCTAAGTTGACTGCCGGTGACAAACCGGAGGAAG

GTGGGGATGACGTCAAATCATCATGCCCCTTATGATTTGGGCTACACACGTGCTACAATGGACAATACAA

AGGGCAGCGAAACCGCGAGGTCAAGCAAATCCCATAAAGTTGTTCTCAGTTCGGATTGTAGTCTGCAACT

CGACTACATGAAGCTGGAATCGCTAGTAATCGTAGATCAGCATGCTACGGTGAATA**CGTTCCCGGGTATT**

**GTACACACCG**CCCGTCACACCACGAGAGTTTGTAACACCCGAAGCCGGTGGAGTAACCTTTTAGGAGCTA

GCCGTCGAAGGTGGGACAAATGATTGGGGTGAAGTCGTAACAAGGTAGCCGTATCGGAAGGTGCGGCTGG

ATCACCTCCTTT

**BURKHOLDERIA CEPACIA ATCC 25416 - 52% GC**

**AGAGTTTGATC**C**TGGCTCAG**ATTGAACGCTGGCGGCATGCCTTACACATGCAAGTCGAACGGCAGCACGGGTGCTTGCACCTGGTGGCGAGTGGCGAACGGGTGAGTAATACATCGGAACATGTCCTGTAGTGGGGGATAGCCCGGCGAAAGCCGGATTAATACCGCATACGATCTACGGATGAAAGCGGGGGACCTTCGGGCCTCGCGCTATAGGGTTGGCCGATGGCTGATTAGCTAGTTGGTGGGGTAAAGGCCTACCAAGGCGACGATCAGTAGCTGGTCTGAGAGGACGACCAGCCACACTGGGACTGAGACACGGCCCAGACTCCTACGGGAGGCAGCAGTGGGGAATTTTGGACAATGGGCGAAAGCCTGATCCAGCAATGCCGCGTGTGTGAAGAAGGCCTTCGGGTTGTAAAGCACTTTTGTCCGGAAAGAAATCCTTGGCTCTAATACAGTCGGGGGATGACGGTACCGGAAGAAAAAGCACCGGCTAACTACGTGCCAGCAGCCGCGGTAATACGTAGGGTGCAAGCGTTAATCGGAATTACTGGGCGTAAAGCGTGCGCAGGCGGTTTGCTAAGACCGATGTGAAATCCCCGGGCTCAACCTGGGAACTGCATTGGTGACTGGCAGGCTAGAGTATGGCAGAGGGGGGTAGAATTCCACGTGTAGCAGTGAAATGCGTAGAGATGTGGAGGAATACCGATGGCGAAGGCAGCCCCCTGGGCCAATACTGACGCTCATGCACGAAAGCGTGGGGAGCAAACAGGATTAGATACCCTGGTAGTCCACGCCCTAAACGATGTCAACTAGTTGTTGGGGATTCATTTCCTTAGTAACGTAGCTAACGCGTGAAGTTGACCGCCTGGGGAGTACGGTCGCAAGATTAAAACTCAAAGGAATTGACGGGGACCCGCACAAGCGGTGGATGATGTGGATTAATTCGATGCAACGCGAAAAACCTTACCTACCCTTGACATGGTCGGAATCCTGCTGAGAGGCGGGAGTGCTCGAAAGAGAACCGGCGCACAGGTGCTGCATGGCTGTCGTCAGCTCGTGTCGTGAGATGTTGGGTTAAGTCCCGCAACGAGCGCAACCCTTGTCCTTAGTTGCTACGCAAGAGCACTCTAAGGAGACTGCCGGTGACAAACCGGAGGAAGGTGGGGATGACGTCAAGTCCTCATGGCCCTTATGGGTAGGGCTTCACACGTCATACAATGGTCGGAGCAGAGGGTTGCCAACCCGCGAGGGGGAGCTAATCCCAGAAAACCGATCGTAGTCCGGATTGCACTCTGCAACTCGAGTGCATGAAGCTGGAATCGCTAGTAATCGCGGATCAGCATGCCGCGGTGAATA**CGTTCCCGGGTCTTGTACACACCG**CCCGTCACACCATGGGAGTGGGTTTTACCAGAAGTGGCTAGTCTAACCGCAAGGAGGACGGTCACCACGGTAGGATTCA

**PSEUDOMONAS AERUGINOSA ATCC 10145 - 54% GC**

**GAGTTTGATCATGGCTCAG**ATTGAACGCTGGCGGCAGGCCTAACACATGCAAGTCGAGCGGATGAAGGGA

GCTTGCTCCTGGATTCAGCGGCGGACGGGTGAGTAATGCCTAGGAATCTGCCTGGTAGTGGGGGATAACG

TCCGGAAACGGGCGCTAATACCGCATACGTCCTGAGGGAGAAAGTGGGGGATCTTCGGACCTCACGCTAT

CAGATGAGCCTAGGTCGGATTAGCTAGTTGGTGGGGTAAAGGCCTACCAAGGCGACGATCCGTAACTGGT

CTGAGAGGATGATCAGTCACACTGGAACTGAGACACGGTCCAGACTCCTACGGGAGGCAGCAGTGGGGAA

TATTGGACAATGGGCGAAAGCCTGATCCAGCCATGCCGCGTGTGTGAAGAAGGTCTTCGGATTGTAAAGC

ACTTTAAGTTGGGAGGAAGGGCAGTAAGTTAATACCTTGCTGTTTTGACGTTACCAACAGAATAAGCACC

GGCTAACTTCGTGCCAGCAGCCGCGGTAATACGAAGGGTGCAAGCGTTAATCGGAATTACTGGGCGTAAA

GCGCGCGTAGGTGGTTCAGCAAGTTGGATGTGAAATCCCCGGGCTCAACCTGGGAACTGCATCCAAAACT

ACTGAGCTAGAGTACGGTAGAGGGTGGTGGAATTTCCTGTGTAGCGGTGAAATGCGTAGATATAGGAAGG

AACACCAGTGGCGAAGGCGACCACCTGGACTGATACTGACACTGAGGTGCGAAAGCGTGGGGAGCAAACA

GGATTAGATACCCTGGTAGTCCACGCCGTAAACGATGTCGACTAGCCGTTGGGATCCTTGAGATCTTAGT

GGCGCAGCTAACGCGATAAGTCGACCGCCTGGGGAGTACGGCCGCAAGGTTAAAACTCAAATGAATTGAC

GGGGGCCCGCACAAGCGGTGGAGCATGTGGTTTAATTCGAAGCAACGCGAAGAACCTTACCTGGCCTTGA

CATGCTGAGAACTTTCCAGAGATGGATTGGTGCCTTCGGGAACTCAGACACAGGTGCTGCATGGCTGTCG

TCAGCTCGTGTCGTGAGATGTTGGGTTAAGTCCCGTAACGAGCGCAACCCTTGTCCTTAGTTACCAGCAC

CTCGGGTGGGCACTCTAAGGAGACTGCCGGTGACAAACCGGAGGAAGGTGGGGATGACGTCAAGTCATCA

TGGCCCTTACGGCCAGGGCTACACACGTGCTACAATGGTCGGTACAAAGGGTTGCCAAGCCGCGAGGTGG

AGCTAATCCCATAAAACCGATCGTAGTCCGGATCGCAGTCTGCAACTCGACTGCGTGAAGTCGGAATCGC

TAGTAATCGTGAATCAGAATGTCACGGTGAATA**CGTTCCCGGGCCTTGTACACACCG**CCCGTCACACCAT

GGGAGTGGGTTGCTCCAGAAGTAGCTAGTCTAACCGCAAGGGGGACGGTTACCACGGAGTGATTCATGAC

TGGGGTGAAGTCGTAACAG

**BACILLUS SUBTILIS NCBI 102783 - 55% GC**

TTATCGG**AGAGTTTGATCCTGGCTCAG**GACGAACGCTGGCGGCGTGCCTAATACATGCAAGTCGAGCGGA

CAGATGGGAGCTTGCTCCCTGATGTTAGCGGCGGACGGGTGAGTAACACGTGGGTAACCTGCCTGTAAGA

CTGGGATAACTCCGGGAAACCGGGGCTAATACCGGATGGTTGTTTGAACCGCATGGTTCAAACATAAAAG

GTGGCTTCGGCTACCACTTACAGATGGACCCGCGGCGCATTAGCTAGTTGGTGAGGTAACGGCTCACCAA

GGCGACGATGCGTAGCCGACCTGAGAGGGTGATCGGCCACACTGGGACTGAGACACGGCCCAGACTCCTA

CGGGAGGCAGCAGTAGGGAATCTTCCGCAATGGACGAAAGTCTGACGGAGCAACGCCGCGTGAGTGATGA

AGGTTTTCGGATCGTAAAGCTCTGTTGTTAGGGAAGAACAAGTGCCGTTCGAATAGGGCGGTACCTTGAC

GGTACCTAACCAGAAAGCCACGGCTAACTACGTGCCAGCAGCCGCGGTAATACGTAGGTGGCAAGCGTTG

TCCGGAATTATTGGGCGTAAAGGGCTCGCAGGCGGTTTCTTAAGTCTGATGTGAAAGCCCCCGGCTCAAC

CGGGGAGGGTCATTGGAAACTGGGGAACTTGAGTGCAGAAGAGGAGAGTGGAATTCCACGTGTAGCGGTG

AAATGCGTAGAGATGTGGAGGAACACCAGTGGCGAAGGCGACTCTCTGGTCTGTAACTGACGCTGAGGAG

CGAAAGCGTGGGGAGCGAACAGGATTAGATACCCTGGTAGTCCACGCCGTAAACGATGAGTGCTAAGTGT

TAGGGGGTTTCCGCCCCTTAGTGCTGCAGCTAACGCATTAAGCACTCCGCCTGGGGAGTACGGTCGCAAG

ACTGAAACTCAAAGGAATTGACGGGGGCCCGCACAAGCGGTGGAGCATGTGGTTTAATTCGAAGCAACGC

GAAGAACCTTACCAGGTCTTGACATCCTCTGACAATCCTAGAGATAGGACGTCCCCTTCGGGGGCAGAGT

GACAGGTGGTGCATGGTTGTCGTCAGCTCGTGTCGTGAGATGTTGGGTTAAGTCCCGCAACGAGCGCAAC

CCTTGATCTTAGTTGCCAGCATTCAGTTGGGCACTCTAAGGTGACTGCCGGTGACAAACCGGAGGAAGGT

GGGGATGACGTCAAATCATCATGCCCCTTATGACCTGGGCTACACACGTGCTACAATGGACAGAACAAAG

GGCAGCGAAACCGCGAGGTTAAGCCAATCCCACAAATCTGTTCTCAGTTCGGATCGCAGTCTGCAACTCG

ACTGCGTGAAGCTGGAATCGCTAGTAATCGCGGATCAGCATGCCGCGGTGAATA**CGTTCCCGGGCCTTGT**

**ACACACCG**CCCGTCACACCACGAGAGTTTGTAACACCCGAAGTCGGTGAGGTAACCTTTTAGGAGCCAGC

CGCCGAAGGTGGGACAGATGATTGGGGTGAAGTCGTAACAAGGTAGCCGTATCGGAAGGTGCGGCTGGAT

CACCTCCTTT

**KLEBSIELLA PNEUMONIAE ATCC 13883 - 56% GC**

**ATCCTGGCTCAG**ATTGAACGCTGGCGGCAGGCCTAACACATGCAAGTCGAGCGGTAGCACAGAGAGCTTG

CTCTCGGGTGACGAGCGGCGGACGGGTGAGTAATGTCTGGGAAACTGCCTGATGGAGGGGGATAACTACT

GGAAACGGTAGCTAATACCGCATAACGTCGCAAGACCAAAGTGGGGGACCTTCGGGCCTCATGCCATCAG

ATGTGCCCAGATGGGATTAGCTAGTAGGTGGGGTAACGGCTCACCTAGGCGACGATCCCTAGCTGGTCTG

AGAGGATGACCAGCCACACTGGAACTGAGACACGGTCCAGACTCCTACGGGAGGCAGCAGTGGGGAATAT

TGCACAATGGGCGCAAGCCTGATGCAGCCATGCCGCGTGTGTGAAGAAGGCCTTCGGGTTGTAAAGCACT

TTCAGCGGGGAGGAAGGCGATGAGGTTAATAACCTCGTCGATTGACGTTACCCGCAGAAGAAGCACCGGC

TAACTCCGTGCCAGCAGCCGCGGTAATACGGAGGGTGCAAGCGTTAATCGGAATTACTGGGCGTAAAGCG

CACGCAGGCGGTCTGTCAAGTCGGATGTGAAATCCCCGGGCTCAACCTGGGAACTGCATTCGAAACTGGC

AGGCTAGAGTCTTGTAGAGGGGGGTAGAATTCCAGGTGTAGCGGTGAAATGCGTAGAGATCTGGAGGAAT

ACCGGTGGCGAAGGCGGCCCCCTGGACAAAGACTGACGCTCAGGTGCGAAAGCGTGGGGAGCAAACAGGA

TTAGATACCCTGGTAGTCCACGCCGTAAACGATGTCGATTTGGAGGTTGTGCCCTTGAGGCGTGGCTTCC

GGAGCTAACGCGTTAAATCGACCGCCTGGGGAGTACGGCCGCAAGGTTAAAACTCAAATGAATTGACGGG

GGCCCGCACAAGCGGTGGAGCATGTGGTTTAATTCGATGCAACGCGAAGAACCTTACCTGGTCTTGACAT

CCACAGAACTTTCCAGAGATGCATTGGTGCCTTCGGGAACTGTGAGACAGGTGCTGCATGGCTGTCGTCA

GCTCGTGTTGTGAAATGTTGGGTTAAGTCCCGCAACGAGCGCAACCCTTATCCTTTGTTGCCAGCGGTTA

GGCCGGGAACTCAAAGGAGACTGCCAGTGATAAACTGGAGGAAGGTGGGGATGACGTCAAGTCATCATGG

CCCTTACGACCAGGGCTACACACGTGCTACAATGGCATATACAAAGAGAAGCGACCTCGCGAGAGCAAGC

GGACCTCATAAAGTATGTCGTAGTCCGGATTGGAGTCTGCAACTCGACTCCATGAAGTCGGAATCGCTAG

TAATCGTAGATCAGAATGCTACGGTGAATA**CGTTCCCGGGCCTTGTACACACCG**CCCGTCACACCATGGG

AGTGGGTTGCAAAAGAAGTAGGTAGCTTAACCTTCG
